# The Psychosocial Experience of Patients with End-Stage Renal Disease and Its Impact on Quality of Life: Findings from a Needs Assessment to Shape a Service

**DOI:** 10.5402/2013/308986

**Published:** 2012-10-21

**Authors:** Jennifer Finnegan-John, Veronica J. Thomas

**Affiliations:** ^1^Florence Nightingale School of Nursing and Midwifery, Kings College London, London SE1 8WA, UK; ^2^Department of Psychology, Institute of Psychiatry, Kings College London, De Crespigny Park, London SE5 8AF, UK; ^3^Department of Haematology, Guy's Hospital, 4th Floor Southwark Wing, Great Maze Pond, London SE1 9RT, UK

## Abstract

*Background*. A needs assessment was conducted on renal patients registered to a leading hospital trust in London in order to explore their psychological, social, and spiritual needs. The aim of the needs assessment was to create an evidence base for the development of a comprehensive health psychology service to run concurrently with a renal counselling support service within the department. *Methodology*. This study utilised a series of semistructured face-to-face interviews and focus groups with renal patients and their carers, to explore how ESRD impacted quality of life. *Results*. A thematic analysis was undertaken. Seven emergent themes were identified that influenced the quality of life of people with EDRD: physiological impact, impact of treatment, impact on daily life, psychological impact, impact on relationships, social impact and coping responses. *Conclusion*. The needs assessment clearly identified that ESRD carries with it emotional, physical, psychological, social, and existential burdens. The data from this needs assessment study has created an evidence base upon which future health psychology services can be built within this leading UK hospital.

## 1. Psychosocial Effects of Kidney Disease

End-stage renal disease (ESRD) is a debilitating, chronic condition whereby the kidney failure requires artificial means of excretion for survival. The primary means to achieve this are by peritoneal dialysis or haemodialysis (done several times weekly). Consequently, patients with ESRD undergo a number of lifestyle, dietary, and fluid restrictions in order to accommodate their illness.

These lifestyle restrictions significantly impact on social functioning with patients performing a balancing act to ensure maintenance of vitamin, iron, and protein levels. Such restrictions can impact on patients' illness beliefs, sense of personal control leading to anxiety and depression, inhibiting coping, and adjustment [1, 2].

Kimmel et al. [1] investigated the impact of psychosocial factors on behavioural compliance and survival in urban haemodialysis patients. Depression was related to decreased adherence to treatment. Depression is strongly recognized as a common psychological problem in haemodialysis patients [3–6]. The rate of psychiatric disorders in a population of ESRD patients was considerably higher than in a population with other chronic medical conditions [3]. Chilcot et al. [7] reported that 20–30% of ESRD patients have significant depressive symptoms compared to the lifetime prevalence of depression in the general population of approximately 16% [8]. Depression can complicate long-term conditions, potentially becoming more resistant to treatment over time [1, 3–6]. Depressed patients are found to be three-times as likely to be noncompliant with treatment recommendations as nondepressed patients [9].

Additional stressors associated with ESRD include biochemical imbalance, physiological changes, neurological disturbances, cognitive impairment, and sexual dysfunction. All can potentially play a role in depression.

A qualitative study by Gregory et al. [10] found patients with ESRD receiving haemodialysis developed a new identity and sense of self. This new and evolving emotional/psychological state indicated that patient's became cognisant of a new set of circumstances: an uncertain future, demands of illness, dependence of machinery, medication, and healthcare providers.

Kimmel et al. [1] found that perception of illness intrusion is linked to poorer survival rates. Illness representations predicted nonadherence to fluid restrictions amongst haemodialysis patients [11]. Compared to other chronic illnesses, haemodialysis patients assessed their physical health as markedly diminished [12]. Illness perceptions are significant predictors of coping, adjustment, and outcome [13]. Additionally, within a sample of UK renal patients, patients' perceptions of treatment control predicted survival independently of survival risk factors, including comorbidity, illustrating the negative impact of maladaptive illness perceptions on clinical outcomes [13]. Griva et al. [14] found that treatment and illness perceptions were formed as a function of different ESRD treatment.

Transplanted patients perceived their condition as less chronic, reported fewer symptoms compared to dialysis patients.

Curtin et al. [16] provided insights into “the transformational experience” of survivors of long-term dialysis. The restructuring of illness beliefs and modification of “illness” cognitions result in positive changes; patients can become “active self managers of their disease, its treatment and its manifestations.” “The concept of transformation is often advanced as the embodiment of successful adjustment to a chronic illness” (page 614).

Diminished quality of life (QOL) is linked to limited personal freedom and control, for example, lengthy treatment time extended lives but contributed to these restrictions [3]. Loss of freedom was also associated with protracted preparations for dialysis which was a major source of frustration. Overall, the loss of freedom had wider implications altering marital, family, and social relationships [2].

A sense of control over illness influences coping and adjustment in a long-term physical health problems. Cvengros et al. [17] found that health locus of control could be enhanced by helping patients to focus their attention on practical ways of coping.

A randomised controlled trial of a cognitive behavioural therapy empowerment intervention indicated significant improvements in sense of empowerment, self-care, self-efficacy, and depression, when the following strategies were implemented: identification of problem areas, exploration of emotions associated with these problem areas, goal and strategy development, implementation of behaviour change plans, and stress management. Such interventions might be effective in improving self-management [18].

Impaired self and body image are common psychological consequences of living with ESRD. Self-image and self-esteem have a bearing on aspects of QOL, which encompasses the physical, social, and emotional elements of wellbeing [14]. Patients may have to adjust to their changing appearance by altering the way they dress and how they relate to others.

Dialysis treatment can significantly impact upon body image, as patients might perceive themselves as unattractive [19]. For example, procedures to create a point of access for dialysis via a fistula, neck line, or catheter can all change the appearance of the body. Immunosuppressant drugs taken to prevent organ rejection also contribute to obvious bodily changes impairing self-acceptance. Reflecting upon renal nursing practice, Muringai et al. [20] discussed the need for a “holistic assessment tool” that allows for psychological considerations of body image.

The roles within marital relationships change when a spouse or partner becomes a carer and can give rise to depression in the carer as a consequence. The patient might become the focus of negative emotions [5]. In addition, sexual difficulties can sometimes occur as a result of erectile dysfunction in male patients with ESRD and can undermine intimacy [21, 22]. A literature review by Low et al. [23] explored the impact of caring for a patient with ESRD, in relation to quality of life, psychological health, morbidity, responsibilities or “burdens,” and coping strategies. It concluded the profound changes in roles and relationships on partners and within the family as a consequence of ESRD.

It is apparent from this literature review that a number of disease and treatment related factors that restrict lifestyle and undermine QOL in people with ESRD. Psychological support to help patients cope with lifestyle restrictions and to enhance personal control through self management strategies are essential, as outlined in the National Service Frameworks (Department of Health, 2005) [24].

According to the National Institute of Clinical Excellence (NICE), a “Health needs assessment (HNA) is a systematic method for reviewing the health issues facing a population, leading to agreed priorities and resource allocation that will improve health and reduce inequalities” [25]. The aim of the needs assessment was to create an evidence base for the development of a comprehensive health psychology service to run concurrently with a renal counselling support service within the department as a means of responding to the recommendations made by the National Services Frameworks (NSF) [24].

The purpose of this needs assessment was to investigate ways of improving services to renal patients as part of wider modernisation initiative and to create evidence base of patients needs.

## 2. Participants

118 patients and 12 carers took part in the qualitative phase of this study. Participants from each stage of the renal illness trajectory from predialysis to posttransplantation were represented to explore the range of patient experiences.

## 3. Inclusion Criteria

All adult patients (above 18 years) who currently had or once had a diagnosis of end-stage renal disease and currently received or had received treatment or medical care from the renal services within a single London trust were eligible to participate.

This included the following:

- in patients,
- outpatients,
- transplanted patients.

All dialysis patients (including peritoneal and haemodialysis) were eligible for the needs assessment, including those who were being treated in four satellite units linked to the trust.

## 4. Exclusion Criteria

Patients with recognised mental health difficulties or in the terminal stage of their illness were not included in the study.

## 5. Recruitment Procedure

Fifty patients were recruited to take part in focus group or semistructured interviews at the time of their participation in a brief questionnaire survey element of work that was being conducted internally by the renal department. The remaining 68 patients and 12 carers were recruited by the researcher with the help of doctors and nurses within outpatient's clinics and dialysis units.

Carers were recruited through a process of snowball sampling, whereby some patients who had participated referred interested carers to the researcher.

## 6. Methodology/Procedure

The research utilised a qualitative methodology of semistructured interviews and focus group discussions. Both interview types followed key themes that explored the following:

- Patients experiences of living with ESRD,
- Lifestyle issues relating to living with ESRD,
- Exploration of psychological, spiritual, and emotional needs arising from their illness and treatment.

Specific interview schedules were developed and piloted with two patient representatives, one male post transplant and one female at predialysis stage. Five focus groups were established. Separate groups were arranged for patients and carers/significant others. Approximately 50 individual interviews were arranged at the convenience of the patients.

All patients provided written consent before their interview for interviews and focus groups to be recorded. A thematic analysis was undertaken and qualitative software was (NVivo version 7) used to analyse interview data. Thematic analysis was chosen as most for reporting “experiences, meanings and the reality of participants” [26]. A data driven or inductive approach was taken so that the data was able to speak for itself, encapsulating all the nuances of meaning and language in the context of the renal experience. Consequently, it did not fit into a preexisting coding frame. This method was best at presenting the themes of the whole data set, beyond pure semantic meanings, and looking towards themes that ascertain, explore, and interpret the words of the patients.

## 7. Sample Characteristics

The mean age of the patient sample was 55 years and 40% of the sample was from black and minority ethnic backgrounds. Details of the sample characteristics of patients and carers recruited are illustrated (Table 1). The breakdown of patients recruited to the study across the renal trajectory is shown (Table 2).

## 8. Findings

Analysis of verbatim transcripts identified the following themes.

Physical impact of ESRD

- The impact of treatment, that is, on activities of daily living.
- Psychological impact (loss of control, anxiety and depression).
- Coping responses and the role of religion/spiritual beliefs.
- Impact on relationships.

An important aim of the research was to explore the impact of ESRD on quality of life, hence, an appropriate quality of life framework was used against which the qualitative findings could be contextualised. The World Health Organisation Quality of Life (WHOQOL) model [15] was therefore used for the analysis of the data. The domains and facets which measure overall quality of life and general health are shown (Table 3). The facets in bold were themes that arose from the qualitative data in this analysis.

We have illustrated the interconnected nature the domains of the WHOQOL, and how our data fell into these categories. Additional themes that arose from our data help to contextualise our findings below.

## 9. Physical Impact of ESRD

Fatigue and decreased energy levels significantly impact patients' lives. The quote below illustrates how dialysis-related fatigue has compromised social interactions and family life.

*“Well, I can not do what I used to do. For example, I do not have any social life because I do not have the energy anymore and I get really tired as well. Like before I used to, for example, meet up with my friend and maybe we'd go and visit other people and come in quite late… But now I do not have that energy to stay out that late or to get engaged in any conversations that exert my energy. And like interacting with my baby… When I take her out now, I still have her in the pushchair because although she can walk I cannot manage…” [*F*5]*

Frequent yet necessary visits to the hospital had cumulative effects in terms of fatigue and interfered with normal life.

*“You know you're continuously fatigued, continuously tired… coming up here three times a week, you know, you can't go on holiday and you've got a family and the kids…” [*M*26]*

Numerous patients discussed how physiological changes impacted on their cognitive functioning. The interrelationship between physiological changes and psychological function such as memory and concentration is illustrated.

*“The other thing is your memory goes as well when you're on dialysis, particularly haemo anyway… In fact it's so strange… you read one page and you think “I'm fed up with this” and put it down because you cannot concentrate. I would say that the majority of us have trouble in one way or another with this type of thing.” [*M*66]*

## 10. The Impact of Treatment

Many patients reported their ambivalence towards dependence on dialysis treatment; although essential, they were troubled by its burden on time, quality of life, and sense of control.

*“I hate haemo, absolutely hate it, it's very restricting. When you first start it you can see people around you, how they've been on it for years and how it affects them, you think “now I'm not gonna let it rule my life, I'm not gonna let it take over my life” but within 6 months you can't help that, it's very overwhelming and it does dictate everything you do in your life.” [*F*43]*

The need to have a permanent internal fistula (in the arm) or peritoneal catheter to facilitate treatment brought a double sense of burden; patients had to accommodate the often unsightly aperture in their bodies undermining body image and self-esteem. Many patients talked about feeling uncomfortable displaying their fistula and disguised it under clothing.

Others, who once felt self-conscious about their fistula, over time, accepted its appearance, although they were aware that it attracted attention from the general public.

*“So I do not have a problem with it but I'm aware that other people think about the fistula. It looks like something out of Dr Who, some sort of internal grub.” [*F*7]*

Taking medication was another important part of the treatment regime. Using steroids had duel benefits and adverse side effects that changed bodily appearance. Several patients were unaware of the cumulative effects of steroid use on their appearance.

*“… that was the main impact for me, cos I blew up like a balloon as soon as I started taking steroids… cos I was always really slim, always fit, and healthy, I wore everything that was trendy, and fashionable, and then I put on tons of weight. And I am now walking around in clothes I cannot identify myself in…” [*F*31]*

## 11. Impact on Activities of Daily Living

All of the patients mentioned that the restrictions to fluids, diet, and leisure activities were significant losses. Strict daily restrictions to diet and fluid have to be contended with as constant reminder of the impact of ESRD. Such restrictions also significantly affected social interactions and leisure activities, causing difficulty.

*“The main thing that I suddenly realised is that when you're on dialysis you cannot drink. You can only have 500 mL a day and you suddenly realise that the whole of our social network is based around eating and drinking…. If you go out to someone's house what's the first thing they say? “Would you like a drink?” If you're going to meet a mate, they'll say “we”ll go down the pub”… You're not going to go to a pub and not drink anything at all… And you try going to a restaurant and sticking to diet restrictions, it's very difficult. So all of a sudden you're isolated in that respect. You do not realise it until you cannot do it and that is a massive change.” [M87]*

These restrictions require much in the way of daily forward planning and organisation. Psychological support such as cognitive behaviour therapy might be an effective way of supporting patients that put their health at risk due to poor concordance with fluid restrictions, which over time can result in heart failure.

The physical symptoms of ESRD also affect employment.

*“I feel like I've got no prospects. I cannot get a job. If I go out to look for a job, I get to the interview stage and they start asking me if there are any questions. I say, ‘right, well I'm a kidney patient, I'm waiting for a transplant, I need to do dialysis three times a week which means I need to take x, y, z amount of time off work.” [M98]*

Having to give up employment was another source of stress.

*“I was on dialysis three or four hours a session, three days a week. When I put in for disability allowance they refused me. I had to take them to the tribunal to get disability allowance. I was receiving the treatment at the hospital and still they said I did not qualify.” [M69]*

## 12. Social Activities and Leisure

Patients referred to the time consuming nature of treatment that reduced time for leisure and social activities. Dialysis treatment is intensive physically and emotionally. Patients often dedicate up to three days a week to treatment, and sometimes including the weekend. Treatment sessions can last for several hours at a time, requiring patients to travel to hospital/dialysis satellite unit. The combination of regular travel and lengthy treatment time impacted on personal freedom.

*“You not only lose the whole day that you're here but you lose the rest of the day because you haven't got time in the morning to do anything before you come to the unit, and then you're too tired and not well when you get home at night. So you might as well just write off three days a week.” [F29]*

## 13. Psychological Impact

This theme was an umbrella term which incorporated several subthemes including control over ESRD and ensuing depression.

It appeared that some stages of the disease were more traumatic than others. Some patients experienced psychological trauma around diagnosis or commencement of treatment if they were not well informed about what to expect. These traumas had an impact on their ability to cope with the illness and treatment. A lack of adequate preparation about haemodialysis treatment resulted in a strong psychological response for this patient.

* “… I had a panic attack when they first stuck the needle in [my fistula] because nothing had been explained… and that developed into regular panic attacks…” [M47]*

This contrasted with other patients who received good preparation and education from nursing staff. This was reported as effective for empowering patients, enabling them to cope better.

*“They're very good here. We've all been gradually educated about our dialysis by the nurse in charge… The education was really good I think because it demystifies the whole system [of treatment].” [F37]*

## 14. Self Care and Taking Control

Several patients discussed the importance being given the opportunity to self-care, for example, preparing their machines or “needling” their fistula's for haemodialysis treatment. Patients reported a sense of independence and autonomy which was very important in coping with the routine of treatment.

*“At first it frightened the living daylights out of me when they told me I need to needle myself. But when I've done it I find that I do have more control and it has begun to make me feel a bit more useful… They said they're promoting self-care and I'm all for that. So I'm one of those who is glad that they want us to be involved more and help ourselves more.” [F17]*

The role of the “experienced patient” in empowering newly diagnosed patients was discussed as a positive resource that could provide education and peer support.

*“Patients are your biggest resource. I mean I said this to the doctor. Look, actually your budget is really constrained but did you realise that you've got the biggest resource out there that you're not actually utilising? Your resources are your patients. Utilise their skills and experience and knowledge. Some people will engage with it, some people won't, but patients are such a huge free resource out there. So it's got to be a win-win, has not it?” [F90]*

## 15. Negative Feelings: Depression

In all of the interviews, patients spoke of a variety of losses and treatment burden experienced. Depression was a common psychological response. The following quote describes a loss of freedom.

*“I felt like a bird whose wings had been clipped.” [F40]*

A majority of the patients sampled expressed feelings of depression as a result of reduced quality of life.

*“You cannot help feeling this way. What really depresses me is when I think of other things I probably would have been doing now that I'm unable to do because I'm hooked to the machine. Yes, at times like that I do feel very depressed.” [M80]*

A sense of hopelessness was also expressed.

*“I do not want to sound like I'm suicidal or anything because it's far from but I've been doing this four years now, and I wonder, am I going to get a transplant? I'm thinking to myself can I really do this for another 5 years or 10 years?” [M14]*

In order to cope, most patients reported that they “just got on with it.” For some, feelings of sadness and depression were a constant struggle, and they implied that they felt “trapped” by their circumstances.

## 16. Death and Mortality

Thoughts about dying and death featured in many interviews. Mortality was discussed in relation to withdrawing from treatment. This patient contemplated the idea of withdrawing from dialysis as a “relief” from suffering.

*“Having this renal failure business is so bad. I do not care what happens really. I used to hear about these people signing these forms and two weeks later they're dead. I suppose that's a wonderful relief rather than suffer because I heard they take you off all your medication. You just sign a form and, they stop all your medication, no dialysis, put you on morphine and in two weeks you're a goner.” [F70]*

## 17. Impact on Relationships

Personal relationships were also transformed by the symptoms of ESRD. Fatigue, infertility, low energy, and mood, physical changes to the body, for example, catheter, weight gain, and scarring, all played a role in undermining confidence and self-esteem. Sexual activity and intimacy was also reported as significantly compromised requiring adjustment for both patients and their partners. Changes in perceptions of roles within the relationship were also evident.

*“You still love but its different love… I feel like more of a carer than a wife to be honest or mother even, to some degree. It's very difficult. You just fall into a different role.” [Carer 6]*

Those not currently in a relationship discussed how ESRD might affect their ability to form a relationship.

*“… I would say it's finding the right partner who can cope with someone who's on haemodialysis. At the moment I'm still looking for that partner. It's made me realise that I hope to meet somebody who has an understanding of what I'm going through…” [M100]*

Additional worries were related to sexual dysfunction or an inability to form an intimate relationship. All of these factors combined to diminish confidence.

*“I feel like every time I meet a woman, I feel like I'm broken or there's something missing from me. When I explain about the kidney failure then, I do not know, my confidence just goes down because I feel like I'm not a whole man, there's something wrong with me.” [M107]*

## 18. Availability of Health and Social Care

Patients discussed that they were often in need of additional help after they left hospital. The following statement indicates that there is a significant need for better partnership between health and social care agencies in order to assist chronically ill patients in the best possible way.

*“I think that's what's missing; the hospitals are very good at mending you and sending you out. But the aftercare, like putting you in touch with the right people as you leave hospital, is missing… I think that's where the medical services and the social services should actually get together and make an easier route for us all. We've all had hassle getting help.” [M13]*

Patients also felt that they were disadvantaged by the levels of bureaucracy involved in seeking assistance for social services.

## 19. Coping Responses

Faith and religion was discussed as a means of coping and adjusting to ESRD. Some described how their faith linked them into a religious community, offering a support network. Many said their faith enabled them to keep an existential perspective over their condition. The patient below talked about faith as having a protective effect:

*“Um, my religion and faith has probably stopped me committing suicide.” [*M*90]*

*“… I've seen my faith as a way of supporting the reality of this condition, rather than blaming God for having it.” [*M*7]*

Many patients also discussed how religious beliefs helped them to contextualise their situation; this type of reflection appeared to help some patients focus on “the bigger picture.”

*“Well, there's a God-Without him maybe I wouldn't even be here, I could be dead. So even though I am here in this dialysis chair he's still working with me, he's still there…. I think if you have a faith it all makes sense, it fits into some sort of pattern. If you do not have any faith at all I suppose it must be very frustrating really. But if you have a feeling that it's all part of a big plan then you get on with it I think…” [M27]*

## 20. Discussion

The findings clearly indicate that there was a considerable psychological burden along the renal pathway and considerable impact on quality of life for those living with ESRD. Our sample gave insight into these complexities, as demonstrated by the relationships between domains of the WHOQOL framework [15] and the facets that arose from our data analysis. According to Cukor et al. [27] “stress is a concomitant of chronic illness and its treatment, and may have meaningful influences on psychological or medical outcomes.” The majority of patients in this study reported negative feelings experienced as a result ESRD. Anxiety, depression, and in some cases, suicidal ideation were discussed by the patients in this sample. Some patients also spoke about experiencing grief for the loss of their kidney. This was particularly obvious for those whose transplants had failed. Transplant represents hope for a better quality of life, its failure, coupled with the sense of loss and disappointment as well as a huge sense of dread at having to return to dialysis treatment, is psychologically challenging.

Despite our study being qualitative, the experience of symptoms in our findings comparable to other end-stage patients with advanced cancer, AIDS, heart disease and chronic obstructive pulmonary disease (COPD). The systematic review conducted by Solano et al. [28] showed there was a common pathway of 11 symptoms as prevalent in advanced disease as in advanced cancer. Despite methodological disparities in their included studies, they found that for all 5 diseases, up to 50% of patient experienced pain, breathlessness, and fatigue. Similarly, the systematic review by Janssen et al. [29] also found great heterogeneity of symptoms, they asserted significant symptom burden amongst patients with congestive heart failure, COPD, and chronic renal failure.

Within the renal setting, supporting patients with psychological distress can be addressed by health psychologists, with an aim to improving coping mechanisms and enhancing quality of life. This could be done by establishing services or multidisciplinary care approaches which incorporate a holistic framework.

## 21. Conclusion

Quality of life is defined by the WHOQOL group [30] as “an individual's perception of their position in life in the context of value systems in which they live and in relation to their goals, expectations, standards and concerns.” The data from our study indicates that living with a chronic illness such as end-stage renal disease can greatly compromise the “value systems” that patients hold relating to what gives their lives quality and to what they attribute meaning in their lives. It is clear from those who participated in this study that end-stage renal disease undermines every area of a patient's life.

This study has been valuable in illustrating the ways in which ESRD destabilize QOL. It has also added to existing data, on QOL in renal disease. Additionally, this research has responded to the application qualitative data as a means of informing quality of life within specific illnesses [31]. However, a major limitation of this study is a lack of reference to specific health related quality of life framework and caution should be applied in generalising the present findings to all people with ESRD. NICE guidance on depression in people with physical health problems has huge relevance to ESRD NICE recommends the identification and treatment of depression in adults when such difficulties occur in the context of a chronic health problem [32].

Markers of good practice according to the Department of Health's paper for delivering NSF for renal services [33] are “Referral to a multi-skilled renal team, where possible at least one year before the anticipated start of dialysis treatment, for appropriate clinical and psychological preparation. This principle should also be followed for people with a failing transplant.” Given the range and extent of patient and family members needs identified in this data, a recommended health and social care response, on local and national levels to correspond with NSF standard/quality requirements, would be to provide health psychology support services within hospital and satellite units, providing patient centred care, and supporting patients to manage their condition and “achieve the best quality of life.”

The data from this needs assessment study has created an evidence base upon which future health psychology services can be built within this leading UK hospital. As a direct result of this study, funding was achieved for the employment of a health psychologist and a clinical health psychologist was also appointed to address the huge psychosocial needs of the renal population.

## Figures and Tables

**Table 1:** details of sample characteristics; patients and carers.

	Number of Patients who participated	Number of carers who participated
Males	74	3
Females	44	9

**Table 2:** details of the percentage of patients representing the renal treatment trajectory.

Renal treatment trajectory	% of patients
Pre-dialysis	8
Peritoneal dialysis	4
haemodialysis	54
transplant	31
No disclosure	3

**Table 3:** World health organisation quality of Life assessment; domains of quality of life and the facets that correspond to them; world health organisation, 1997 [15].

Domain	Facet
Physical domain	Pain and discomfort
	**Energy and fatigue**
	Sleep and rest

Psychological Domain	**Positive feeling**
	Thinking, learning, memory, concentration
	**Self esteem**
	**Body image**
	**Negative Feelings **

Level of independence	Mobility
	**Activities of daily living**
	**Dependence on medication or treatments**
	**Working capacity **

Social relationships	**Personal relationships**
	**Social support**
	**Sexual activity **

Environment	Physical safety
	Home environment
	Financial resources
	**Health and Social Care: availability and quality**
	**Opportunities for acquiring new** **information and skills**
	Participation in and opportunities for leisure
	Physical environment
	Transport

Spirituality	**Religious/personal beliefs**
